# Reduced default mode network connectivity relative to white matter integrity is associated with poor cognitive outcomes in patients with idiopathic normal pressure hydrocephalus

**DOI:** 10.1186/s12883-021-02389-0

**Published:** 2021-09-13

**Authors:** Shigenori Kanno, Kun-ichi Ogawa, Hiroaki Kikuchi, Masako Toyoshima, Nobuhito Abe, Kazushi Sato, Koichi Miyazawa, Ryuji Oshima, Satoru Ohtomo, Hiroaki Arai, Satoshi Shibuya, Kyoko Suzuki

**Affiliations:** 1 Department of Behavioral Neurology and Cognitive Neuroscience, Tohoku University Graduate School of Medicine, 2-1, Seiryo-machi, Aoba-ku, Miyagi 980-8575 Sendai, Japan; 2 Department of Neurology, South Miyagi Medical Center, Shibata, Japan; 3 Department of Radiology, South Miyagi Medical Center, Shibata, Japan; 4 Healthcare Center, South Miyagi Medical Center, Shibata, Japan; 5 Department of Rehabilitation, South Miyagi Medical Center, Shibata, Japan; 6 Kokoro Research Center, Kyoto University, Kyoto, Japan; 7 Department of Neurology, Tohoku Medical and Pharmaceutical University, Sendai, Japan; 8 Department of Neurosurgery, South Miyagi Medical Center, Shibata, Japan; 9 Department of Neurology, Moriyama Memorial Hospital, Edogawa, Japan

**Keywords:** Alzheimer’s disease, Default mode network, Diffusion tensor imaging, Functional magnetic resonance imaging, Idiopathic normal pressure hydrocephalus, Rey auditory verbal learning test.

## Abstract

**Background:**

The aim of this study was to investigate whether default mode network (DMN) connectivity and brain white matter integrity at baseline were associated with severe cognitive impairments at baseline and poor cognitive outcomes after shunt placement in patients with idiopathic normal pressure hydrocephalus (iNPH).

**Methods:**

Twenty consecutive patients with iNPH whose symptoms were followed for 6 months after shunt placement and 10 healthy controls (HCs) were enrolled. DMN connectivity and brain white matter integrity at baseline in the patients with iNPH and HCs were detected by using resting-state functional magnetic resonance imaging (MRI) with independent component analysis and diffusion tensor imaging, respectively, and these MRI indexes were compared between the patients with iNPH and HCs. Performance on neuropsychological tests for memory and executive function and on the gait test was assessed in the patients with iNPH at baseline and 6 months after shunt placement. We divided the patients with iNPH into the relatively preserved and reduced DMN connectivity groups using the MRI indexes for DMN connectivity and brain white matter integrity, and the clinical measures were compared between the relatively preserved and reduced DMN connectivity groups.

**Results:**

Mean DMN connectivity in the iNPH group was significantly lower than that in the HC group and was significantly positively correlated with Rey auditory verbal learning test (RAVLT) immediate recall scores and frontal assessment battery (FAB) scores. Mean fractional anisotropy of the whole-brain white matter skeleton in the iNPH group was significantly lower than that in the HC group. The reduced DMN connectivity group showed significantly worse performance on the RAVLT at baseline and significantly worse improvement in the RAVLT immediate recall and recognition scores and the FAB scores than the preserved DMN connectivity group. Moreover, the RAVLT recognition score highly discriminated patients with relatively preserved DMN connectivity from those with relatively reduced DMN connectivity.

**Conclusions:**

Our findings indicated that iNPH patients with reduced DMN connectivity relative to the severity of brain white matter disruption have severe memory deficits at baseline and poorer cognitive outcomes after shunt placement. However, further larger-scale studies are needed to confirm these findings.

**Supplementary Information:**

The online version contains supplementary material available at 10.1186/s12883-021-02389-0.

## Introduction

Idiopathic normal pressure hydrocephalus (iNPH) is a neurological syndrome that carries no causative antecedent disease and is characterised by the clinical triad of gait disturbance, cognitive impairment, and urinary incontinence [1, 2]. The prevalence of the disease, based on the criteria of the Japanese Guidelines for iNPH [3–5], is between 0.51 and 2.9 % [6–8]. Because iNPH is a common disease in elderly individuals and has been shown to be treatable with cerebrospinal fluid (CSF) shunt placement, an early and accurate diagnosis of iNPH has become increasingly important [1, 9]. Although the presence of disproportionately enlarged subarachnoid space hydrocephalus (DESH), especially the finding of high-convexity tightness, on magnetic resonance imaging (MRI) or computed tomography highly predicts shunt responsiveness [1, 10], recent studies have reported that the presence of Alzheimer’s disease (AD) pathology is related to worse baseline cognitive performance and diminished postoperative outcomes in patients with iNPH [11, 12]. Brain amyloid imaging agents, such as [^11^ C]Pittsburgh compound-B and [^18^ F]flutemetamol, are useful tools for detecting the presence of amyloid-β pathology in preoperative patients with iNPH [13, 14]. However, these agents are remarkably expensive and cannot be used for clinical examination in the Japanese health insurance system.

In our previous study, we used diffusion tensor imaging (DTI) to demonstrate that the brain white matter is more involved in patients with iNPH than in patients with AD [15]. Although we compared preoperative DTI measures of brain white matter, such as fractional anisotropy (FA) and mean diffusivity (MD), between shunt-responsive and non-shunt-responsive patients with iNPH in our earlier study, there were no significant differences in these measures between the groups [16]. The presence of AD pathology may not strongly affect the preoperative FA and MD values in patients with iNPH. Although recent DTI studies have reported tau-related white matter alterations in patients with mild cognitive impairment or AD, severe changes in brain white matter integrity by ventricular dilation may obscure white matter alterations due to the presence of AD pathology in patients with iNPH [17, 18].

In recent studies, resting-state functional MRI has been used to investigate synchronous activations occurring at rest among spatially distinct regions [19, 20]. Resting-state functional MRI focuses on spontaneous low-frequency fluctuations in blood oxygenation level-dependent signals and can be used to identify various resting-state brain networks. The default mode network (DMN) is thought to be the most fundamental resting-state brain network and involves the medial prefrontal cortex, posterior cingulate cortex, precuneus, inferior parietal lobe, lateral temporal cortex, hippocampus, and parahippocampal gyrus [21, 22]. The DMN is consistently activated during rest but not during cognitive tasks (task negative) and is thought to be associated with monitoring the internal and external environment and memory [23–26]. In addition, the DMN Khoo et al. recently demonstrated that connectivity within the DMN as measured by resting-state functional MRI is altered and that reduced DMN functional connectivity is strongly associated with executive dysfunction in patients with iNPH [27]. Furthermore, hypoconnectivity within the DMN has also been observed in normal ageing and patients with mild cognitive impairment and AD; in the latter, it has been also reported to be correlated with memory deficits [28, 29]. The precuneus and posterior cingulate cortex are thought to play a key role in controlling the DMN, and hypometabolism or hypoperfusion in these areas is a characteristic feature of patients with AD [30, 31]. Therefore, we hypothesised that reduced DMN connectivity relative to the severity of brain white matter disruption would be related to severe cognitive deficits at baseline and poor cognitive outcomes after shunt placement in patients with iNPH.

The aim of this study was to investigate whether iNPH patients with severe cognitive impairments and poor cognitive outcomes after shunt placement could be detected using indexes of both DMN connectivity and brain white matter integrity at baseline. For this purpose, we used resting-state functional MRI and DTI data to measure DMN connectivity and brain white matter integrity, respectively. Furthermore, we investigated whether preoperative clinical measures could serve as substitutes for the role of these MRI indexes in detecting iNPH patients with poor cognitive outcomes after shunt placement.

## Methods

### Participants

Twenty (8 women/12 men) consecutive patients with iNPH who underwent shunt surgery at South Miyagi Medical Center were enrolled in this study from February 2017 to June 2018. The patients were diagnosed by board-certified neurologists based on the diagnostic criteria established in accordance with the second edition of the Japanese Clinical Guidelines for iNPH [3–5]. The inclusion criteria for iNPH patients in this study were as follows: (1) > 60 years of age; (2) gait disturbance, dementia, and/or urinary incontinence; (3) ventricular dilatation (Evans index > 0.3) with a narrow CSF space in the superior convexity (i.e., DESH); (4) CSF pressure < 200 mm H_2_O with normal CSF cell counts and protein levels; (5) the absence of other diseases that may account for such symptoms; and (6) the absence of a history of illness that may have caused ventricular dilatation. In addition, 10 healthy controls (HCs) who underwent medical check-ups of the brain at South Miyagi Medical Center were enrolled. The inclusion criteria for the HCs were as follows: (1) > 60 years of age; (2) absence of a history of illness that may have caused motor, cognitive, and/or urinary dysfunction; and (3) absence of abnormal findings on neurological examination or brain MRI. The demographic characteristics of the participants are shown in Table 1. The mean age of the iNPH group was significantly higher than that of the HC group.

**Table 1:** Demographic and clinical characteristics of the participants

Variables	iNPH (*n* = 20)	HCs (*n* = 10)	*P* value
Age (years)	79.4 ± 6.1	70.2 ± 7.3	0.001
Sex (women/men)	8/12	4/6	0.259
Educational attainment (years)	10.6 ± 3.1	12.1 ± 1.7	0.093
Disease duration (years)	2.4 ± 1.8		
iNPHGS			
Gait	2.0, 0–3		
Cognition	2.0, 1–3		
Urination	1.5, 0–3		
Total	5.5, 2–8		
MMSE (/30)	23.2 ± 5.0	28.1 ± 2.2	0.314
FAB (/18)	11.9 ± 2.8	16.2 ± 1.5	0.004
RAVLT			
Immediate recall (/75)	22.2 ± 9.0	39.3 ± 8.6	0.006
Delayed recall (/15)	2.3 ± 2.2	8.8 ± 2.0	< 0.001
Recognition (/30)	24.0 ± 4.4	27.9 ± 1.1	0.111
TUG			
Completion time (seconds)	17.6 ± 13.1	N/A	
Number of steps	28.9 ± 18.8	N/A	
DMN connectivity	2.751 ± 0.441	3.857 ± 1.191	0.018
ROI FA	0.459 ± 0.021	0.481 ± 0.012	0.018

Data are presented as means ± SDs except for sex (women/men) and iNPHGS scores (median, range). Analysis of covariance used except for age (Student’s t-test), sex (chi-square test), and educational attainment (Student’s t-test)

*DMN* default mode network, *FAB* frontal assessment battery, *HCs* healthy controls, *iNPH* idiopathic normal pressure hydrocephalus, *iNPHGS* idiopathic normal pressure hydrocephalus grading scale, *MMSE* Mini-Mental State Examination, *N/A* not assessed, *RAVLT* Rey auditory verbal learning test, *ROI FA* mean fractional anisotropy value within the whole-brain white matter region of interest, *TUG *Timed Up and Go test

All patients underwent a ventriculoperitoneal (VP) shunt procedure. Shunt implantation was conducted using a Codman-Hakim programmable valve with a Siphon-Guard (Codman and Shurtleff, Johnson and Johnson Inc, Raynham, MA, USA). A shunt tube was inserted in the right anterior horn of the lateral ventricle. Postoperatively, the patients were followed at the outpatient clinic, and the pressure setting of their programmable values was adjusted in a stepwise manner. Pressure adjustments were made repeatedly until the optimal pressure for each patient was attained. All patients were re-evaluated approximately 6 months after shunt surgery. Fourteen (7 women/7 men) patients showed significant shunt responsiveness, which is defined as an improvement by one or more points on the total score on the idiopathic normal pressure hydrocephalus grading scale (iNPHGS) [32].

### Clinical assessments

In the present study, clinical measures were assessed prior to performing both CSF removal and shunt placement and re-assessed approximately 6 months after shunt placement. In addition to the iNPHGS, we administered the Timed Up and Go test (TUG) [33] to evaluate gait function and a series of standard neuropsychological tests, including the Mini-Mental State Examination (MMSE) [34], the frontal assessment battery (FAB) [35], and the Rey auditory verbal learning test (RAVLT) [36]. The FAB is a simple and well-established test battery for assessing executive/frontal lobe function. It includes six subtests that assess the following aspects: (1) conceptualisation and abstract reasoning (similarities); (2) mental flexibility (phonemic verbal fluency); (3) motor programming and executive control of action (Luria motor sequences); (4) resistance to interference (conflicting instructions); (5) self-regulation and inhibitory control (go/no-go test); and (6) environmental autonomy (prehensive behaviour). Each subtest is scored from 0 to 3. The RAVLT is commonly used to assess verbal learning and memory. First, 15 words (List A) were read aloud with a one-second interval between each word for five consecutive trials. The participants were asked to repeat as many words as possible in each trial. After the fifth trial, 15 different words (List B) were read aloud, and participants were asked to repeat as many words in List B as possible. Immediately afterward, the participants are asked to recall the words in List (A) After a 20-min delay period, the subjects are asked to recall the words in List A again, and immediately after that, they are required to select the words that they had heard from a 30-word list, which included the 15 initially presented words (List A) and 15 distractor words that were different from the words on List (B) We used the total number of correct answers from the first five trials as the immediate recall score, the number of correct answers from the 20-min delayed recall trial as the delayed recall score, and the total number of true positive and true negative responses from the 20-min delayed recognition trial as the recognition score.

We used the FAB and RAVLT because executive dysfunction is a characteristic feature of cognitive impairment in patients with iNPH, and memory deficits are core symptoms in patients with AD [37, 38].

### MRI Procedure

Cranial MRI was performed prior to performing both CSF removal and shunt placement using a GE Signal 3-Tesla MRI unit (General Electric Company, Milwaukee, WI, USA). Functional imaging and DTI data were acquired using a single-shot spin-echo echo-planar imaging sequence. The imaging parameters used for the acquisition of the axial functional imaging data were repetition time (TR) = 2,500 ms, echo time (TE) = 30 ms, flip angle = 90 degrees, 3.2-mm slice thickness with 0.8-mm insertion gap for 40 slices, field of view (FOV) = 212 × 212 mm^2^, and matrix = 64 × 64. A continuous resting-state scan was performed for approximately 6 min. The subjects were instructed to relax, lie still, and think about nothing as much as possible in the scanner while keeping their eyes open and remaining awake [39]. One hundred forty-four brain volumes were obtained for each subject [40]. The first four scans were discarded to avoid the influence of magnetization instability.

The imaging parameters used for the acquisition of the DTI data were TR = 10,000 ms, TE = 95.2 ms, 2-mm slice thickness with no insertion gap for 75 slices, FOV = 256 × 256 mm^2^, and matrix = 256 × 256. Axial diffusion-weighted imaging data were acquired along 30 gradient directions with b = 1000 s/mm^2^. One volume was acquired without diffusion weighting (b = 0 s/mm^2^).

In addition, high-resolution structural image data were acquired using a three-dimensional spoiled gradient echo (3D-SPGR) imaging sequence. The imaging parameters used for the acquisition of the sagittal 3D-SPGR images were TR = 8.7 ms, TE = 3.2 ms, 1.0-mm slice thickness with no insertion gap for 176 slices, FOV = 256 × 256 mm^2^, and matrix = 256 × 256 (1-mm isotropic voxels).

### Functional image data processing

Functional image data processing was performed according to the method in Khoo et al.’s study [27]. Structural images of the skull, dura, and sinuses obtained via 3D-SPGR imaging were manually stripped using MRIcron [41] and Wacom™ tablets (Cintiq 12WX). We used Multivariate Exploratory Linear Optimized Decomposition into Independent Components (MELODIC) from the FSL software package (https://fsl.fmrib.ox.ac.uk/fsl/fslwiki) to analyse the functional images [42]. The functional images of each subject were motion corrected, and the nonbrain structures were removed. These processed images were then smoothed using a Gaussian kernel with a 5-mm full-width at half-maximum and temporally filtered with a high-pass filter with a cut-off frequency of 0.01 Hz. The functional images were registered to the individual’s skull-stripped structural images and then normalised to the older adult–based Montreal Neurological Institute 152 standard space image [43] using FMRIB’s Nonlinear Image Registration Tool. These normalised data were used for independent component analysis (ICA) to identify large-scale patterns of functional connectivity in each group. We applied a probabilistic principal component model to estimate the optimal number of components. The normalised functional datasets were decomposed into 67 components in the iNPH group and 44 components in the HC group.

**Fig. 1:**
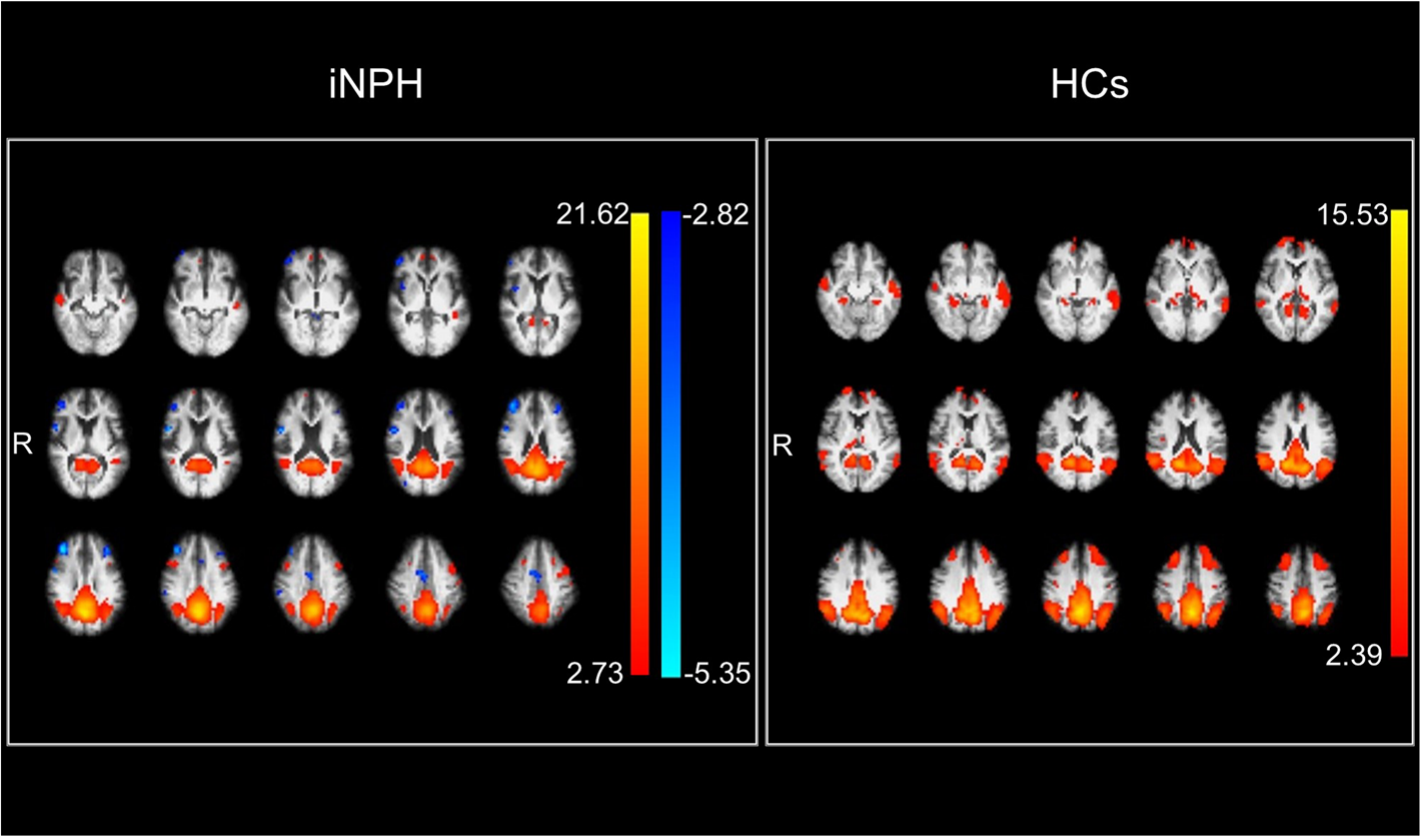
Default mode network detected by group independent component analysis. Left: patients with idiopathic normal pressure hydrocephalus. Right: healthy controls. The coloured bars (red-yellow and sky blue-blue) indicate z-values. iNPH: idiopathic normal pressure hydrocephalus; HCs: healthy controls; R: right

The component that most closely matched the DMN was selected using an automated 2-step process called the “goodness-of-fit” approach for each group. We used Map 4_20,_ which was included among the 20-dimensional ICA and BrainMap components (http://fsl.fmrib.ox.ac.uk/analysis/brainmap+rsns/), as the standard DMN template for selecting the best fitting component in each group. The selected component for each group is presented in Fig. 1. The dual-regression approach was used to identify subject-specific time courses and spatial maps of the DMN, and the intensity of a voxel in the spatial map for each subject represented the degree to which the time series of the voxel correlated with those of the DMN [44]. Moreover, Fisher’s Z-transformation was used to compare the correlation coefficients of DMN connectivity between the iNPH and HC groups. One-sample t-tests in the SPM12 software package (The Wellcome Centre for Human Neuroimaging, UCL Queen Square Institute of Neurology, London, UK) were performed to create a DMN region of interest (ROI) using the spatial maps for each group. We used a voxel-based threshold of *p* < 0.001 (uncorrected) and a cluster-based threshold of *p* < 0.05 (family-wise error corrected) for significance. The mean intensity of the voxels in the DMN ROI across subjects within each group was used as a measure of DMN connectivity [27].

### Diffusion tensor image data processing

**Fig. 2:**
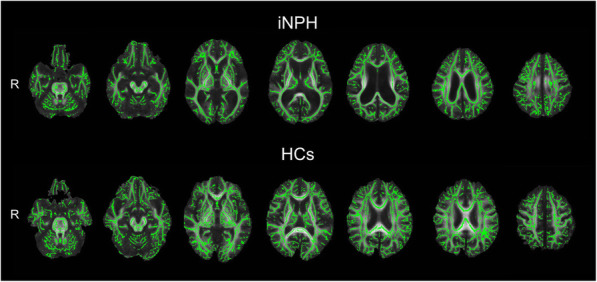
Mean fractional anisotropy skeletons of whole-brain white matter tracts. Top: patients with idiopathic normal pressure hydrocephalus. Bottom: healthy controls. The skeleton (green) of each group is overlaid with the mean fractional anisotropy image of each group. iNPH: idiopathic normal pressure hydrocephalus; HCs: healthy controls; R: right

All diffusion images for each subject were aligned with the initial b0 image using motion correction and registration software (eddy current correction) from the FSL software package. FA maps were calculated from the diffusion-weighted images for each direction using DTI calculation software from the FSL software package (DTIFIT Reconstruct diffusion tensors software). To create a whole-brain white matter ROI, we used algorithms from tract-based spatial statistics (TBSS) [45]. The initial step of TBSS consisted of determining the most representative FA map (the most typical map among the subjects in the analysed group) as the one needing the least warping for all the other maps to align to it. This map was used as the target image, and the FA maps of the subjects were nonlinearly transformed into the space of the target image for each group. The transformed FA maps were averaged to create a mean FA skeleton of the whole white matter tracts using an algorithm that determined the local FA maxima along the perpendicular direction of a particular white matter tract. An FA threshold of 0.2 was then used to differentiate between grey and white matter. We used the voxels within the mean FA skeleton in which the FA values were above 0.2 as the whole-brain white matter ROI (Fig. 2). Each subject’s warped FA map was projected onto the mean FA skeleton for each group, and the final images were normalised to the Montreal Neurological Institute standard space. The mean FA value within the whole-brain white matter ROI (ROI FA) was calculated as a measure of brain white matter integrity for each subject. In addition, the DMN/FA ratio, which was calculated by dividing the DMN connectivity by the ROI FA, was defined as the index reflecting the degree to which DMN connectivity was preserved or reduced with respect to the severity of brain white matter disruption.

### Statistical analyses and classification into preserved and reduced DMN connectivity groups

Cognitive scores, including the MMSE, FAB, and RAVLT immediate recall, delayed recall, and recognition scores, DMN connectivity, and ROI FA, were compared between the iNPH and HC groups using analysis of covariance controlling for age because not only cognitive functions but also DMN connectivity and FA values in brain white matter regions have been reported to be associated with ageing [46–48]. In addition, Pearson’s partial correlation coefficients corrected for age with Bonferroni correction were calculated to identify potential associations between DMN connectivity and the cognitive measures (FAB scores and RAVLT immediate recall, delayed recall, and recognition scores), except for the iNPHGS cognitive subscores (for which Spearman’s partial rank correlation coefficient was calculated), between ROI FA and these variables, and between DMN connectivity and ROI FA in the iNPH group.

**Fig. 3:**
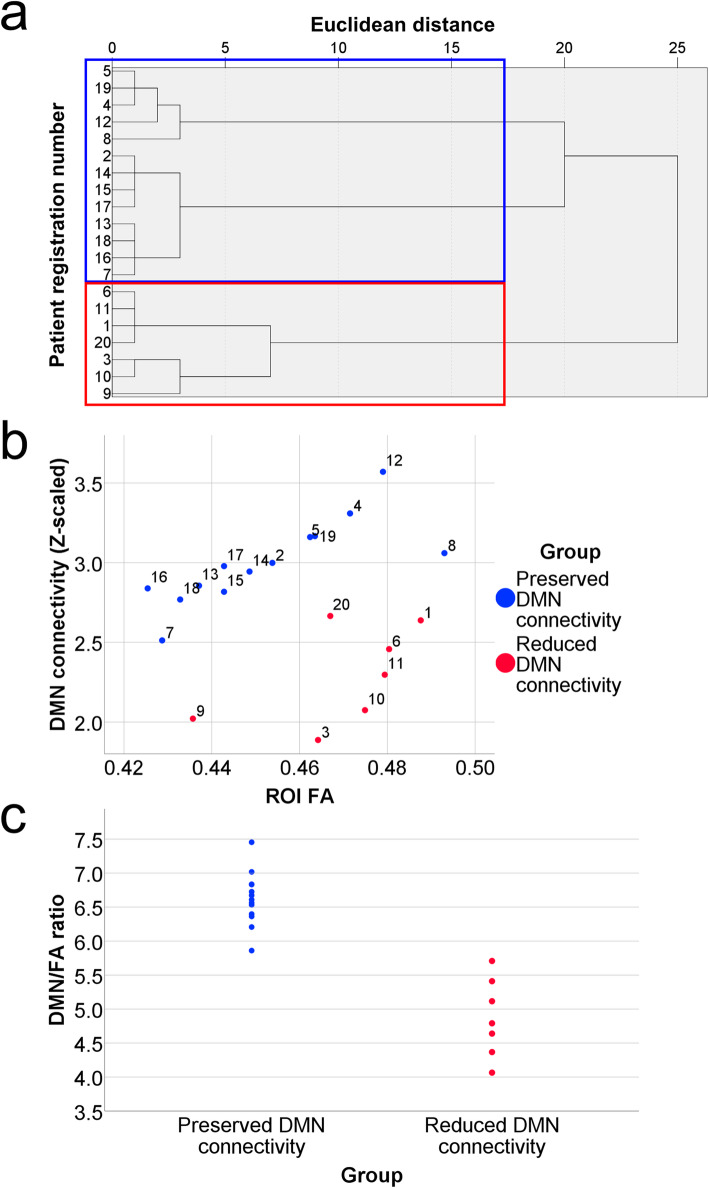
Results of hierarchical clustering and associations between default mode network connectivity and white matter integrity. **a** shows the hierarchical clustering results with Ward’s agglomerative method. The patients were divided into two groups of 13 (blue frame) and 7 patients (red frame). **b** shows a scatter plot between the default mode network (DMN) connectivity and mean fractional anisotropy value within the whole-brain white matter region of interest (ROI FA) of the patients. Blue circles indicate the patients with preserved DMN connectivity relative to the ROI FA (preserved DMN connectivity group). Red circles indicate the patients with reduced DMN connectivity relative to the ROI FA (the reduced DMN connectivity group). The numbers near the circles indicate patient registration numbers. **c** shows dot plots of the DMN connectivity to ROI FA (DMN/FA) ratio in the preserved and reduced DMN connectivity groups. DMN: default mode network; DMN/FA: default mode network connectivity/mean fractional anisotropy value within the brain white matter region of interest; ROI FA: mean fractional anisotropy value within the whole-brain white matter region of interest

To classify the patients with iNPH into two groups based on DMN connectivity and ROI FA, hierarchical clustering with Ward’s agglomerative method was used. Then, these patients were divided into two groups of 13 patients and 7 patients (Fig. 3-a and -b). Because the DMN/FA ratios of the 13 patients were all higher than those of the 7 patients, we labelled the 13 patients as the preserved DMN connectivity group and the 7 patients as the reduced DMN connectivity group (Fig. 3-c). The differences that were observed regarding age, educational attainment, disease duration, iNPHGS total score and subscores, TUG test completion time and number of steps, MMSE score, FAB score, and RAVLT immediate, delayed recall, and recognition scores between the preserved and reduced DMN connectivity groups at baseline were examined using the Mann-Whitney U test, whereas the chi-square test was used for comparisons of sex and shunt responsiveness. In addition, the Mann-Whitney U test and the Wilcoxon signed-rank test were used to evaluate the changes in these clinical measures after shunt placement between the preserved and reduced DMN connectivity groups and within each patient group, respectively. Moreover, receiver operating characteristic (ROC) curves were used to determine whether these two patient groups could be distinguished using the clinical measures at baseline in a manner that revealed significantly worse performance in the reduced DMN connectivity group. Statistical analyses were performed using IBM SPSS statistics software (version 25.00; IBM SPSS Inc., Armonk, NY, USA), and statistical significance was defined as *p*-values < 0.05.

## Results

### Patients with iNPH versus HCs

Comparisons of the clinical and MRI measures between the iNPH and HC groups are presented in Table 1. The mean FAB, and RAVLT immediate and delayed recall scores in the iNPH group were significantly lower than those in the HC group (FAB, [F(1, 27) = 10.184, *p* = 0.004]; RAVLT immediate recall, [F(1, 27) = 9.095, *p* = 0.006]; and RAVLT delayed recall, [F(1, 27) = 31.960, *p* < 0.001]), whereas there was no significant difference in the mean MMSE or RAVLT recognition score between the two groups (MMSE, [F(1, 27) = 1.052, *p* = 0.314] and RAVLT recognition, [F(1, 27) = 2.720, *p* = 0.111]). DMN connectivity in the iNPH group was significantly reduced relative to that in the HC group (DMN connectivity, [F(1, 27) = 6.287, *p* = 0.018]), and the ROI FA in the iNPH group was significantly lower than that in the HC group (ROI FA, [F(1, 27) = 6.381, *p* = 0.018]).

### Correlation analyses within patients with iNPH

The results of the correlation analyses with Bonferroni correction (significance defined as *p*-values < 0.05/5), with the exception of the correlation between DMN connectivity and ROI FA, are shown in Table 2. In the iNPH group, DMN connectivity was significantly positively associated with FAB scores and RAVLT immediate recall scores and was negatively associated with iNPHGS cognitive subscores. There were no significant correlations between DMN connectivity and RAVLT delayed recall and recognition scores. In addition, ROI FA values were not significantly correlated with FAB scores, RAVLT immediate recall, delayed recall, or recognition scores or iNPHGS cognition subscores in the iNPH group. Moreover, there was no significant correlation between DMN connectivity and ROI FA (*p* = 0.797).

**Table 2:** Results of correlation analyses in the iNPH group (*n* = 20)

Variables	DMN connectivity	ROI FA
iNPHGS cognitive	r_s_ = -0.576, *p* = 0.010	r_s_ = -0.040, *p* = 0.872
FAB	*r* = 0.613, *p* = 0.005	*r* = 0.372, *p* = 0.117
RAVLT immediate recall	*r* = 0.667, *p* = 0.002	*r* = -0.160, *p* = 0.512
RAVLT delayed recall	*r* = 0.510, *p* = 0.026	*r* = -0.054, *p* = 0.826
RAVLT recognition	*r* = 0.552, *p* = 0.014	*r* = -0.290, *p* = 0.229

Pearson’s partial correlation coefficient corrected for age with Bonferroni correction was used except for iNPHGS cognitive subscores (Spearman’s partial rank correlation coefficient). Statistical significance was defined as *p*-values < 0.05/5

*DMN* default mode network, *FAB* frontal assessment battery, *iNPH* idiopathic normal pressure hydrocephalus, *iNPHGS* idiopathic normal pressure hydrocephalus grading scale, *ROI FA* mean fractional anisotropy value within the brain white matter region of interest

### Patients with preserved versus reduced DMN connectivity

Comparisons of the baseline demographic and clinical and MRI measures between the preserved and reduced DMN connectivity groups are shown in Table 3. There were no significant differences between DMN connectivity groups in age, sex ratio, disease duration, educational attainment, iNPHGS total scores and gait and urination subscores, MMSE and FAB scores, and TUG test completion times and number of steps, whereas iNPHGS cognition subscores and RAVLT immediate recall, delayed, and recognition scores were significantly worse in the reduced DMN connectivity group than in the preserved DMN connectivity group.

**Table 3:** Comparison of data at baseline between the preserved and reduced DMN connectivity groups

Variables	Preserved DMN connectivity group (*n* = 13)	Reduced DMN connectivity group (*n* = 7)	*P* value
Age (years)	80.0, 65–89	80.0, 69–85	0.877
Sex (women/men)	5/8	3/4	0.848
Disease duration (years)	2.7, 0.2–5.6	1.8, 0.1–5.1	0.211
Educational attainment (years)	9.0, 8–18	9.0, 6–12	0.241
Responder/non-responder	11/2	3/4	0.052
iNPHGS			
Gait	2.0, 1–3	2.0, 0–3	0.817
Cognition	2.0, 1–3	3.0, 2–3	0.046
Urination	1.0, 0–3	2.0, 0–3	0.536
Total	5.0, 2–8	7.0, 2–8	0.183
MMSE (/30)	25.0, 13–29	23.0, 11–27	0.135
FAB (/18)	13.0, 9–15	11.0, 5–16	0.275
RAVLT			
Immediate recall (/75)	25.0, 14–41	18.0, 6–24	0.008
Delayed recall (/15)	2.0, 0–9	0.0, 0–3	0.046
Recognition (/30)	27.0, 20–30	21.0, 14–25	0.003
TUG			
Completion time (seconds)	12.4, 7.2–53.7	16.9, 8.4–50.2	0.485
Number of steps	22.0, 14–81	21.0, 16–62	0.699
DMN functional connectivity	2.978, 2.512–3.570	2.297, 1.887–2.665	< 0.001
ROI FA	0.449, 0.425–0.493	0.475, 0.436–0.488	0.056
DMN/FA	6.608, 5.860–7.454	4.791, 4.065–5.708	< 0.001

Data are presented as medians and ranges except for sex (women/men) and shunt responsiveness (responder/non-responder). Mann-Whitney U tests were used except for sex and shunt responsiveness (chi-square test)

*DMN* default mode network, *DMN/FA* default mode network connectivity/ mean fractional anisotropy value within the brain white matter region of interest, *FAB* frontal assessment battery; HCs: healthy controls, *iNPH* idiopathic normal pressure hydrocephalus, *iNPHGS* the idiopathic normal pressure hydrocephalus grading scale, *MMSE* Mini-Mental State Examination, *RAVLT* Rey auditory verbal learning test, *ROI FA* mean fractional anisotropy value within the brain white matter region of interest, *TUG* Timed Up and Go test

Table 4 shows the changes in clinical measures after shunt placement in the preserved and reduced DMN connectivity groups. According to the results of the Mann-Whitney U test, FAB and RAVLT immediate recall and recognition scores in the preserved DMN connectivity group improved to a significantly greater extent than those in the reduced DMN connectivity group. On the other hand, there were no significant differences in iNPHGS total scores and all subscores, MMSE scores, RAVLT delayed recall scores, or TUG test completion times or number of steps between the two groups. The Wilcoxon signed-rank test revealed significant improvements after shunt placement in all clinical measures we examined except for the iNPHGS cognition score in the preserved DMN connectivity group. In contrast, there were no significant improvements after shunt placement in any of the clinical measures we examined except for the iNPHGS gait subscore in the reduced DMN connectivity group.

**Table 4:** Clinical outcomes after shunt placement in patients with iNPH

Variables	Preserved DMN connectivity group (*n* = 13)				Reduced DMN connectivity group (*n* = 7)				Between-group comparison of change
	Baseline	Post-op	Change	*P* value	Baseline	Post-op	Change	*P* value	*P* value
iNPHGS									
Gait	2.0, 1–3	1.0, 0–2	-1.0, -1-0	0.008	2.0, 0–3	1.0, 0–2	-1.0, -1-0	0.046	0.938
Cognition	2.0, 1–3	2.0, 1–2	0.0, -1-0	0.083	3.0, 2–3	3.0, 2–3	0.0, 0–0	1.000	0.327
Urination	1.0, 0–3	1.0, 0–2	0.0, -1-0	0.014	2.0, 0–3	2.0, 0–3	0.0, -1-1	1.000	0.519
Total	5.0, 2–8	4.0, 2–6	-1.0, -2-0	0.002	7.0, 2–8	7.0, 2–8	0.0, -2-0	0.102	0.281
MMSE (/30)	25.0, 13–29	28.0, 18–30	2.0, 0–5	0.003	23.0, 11–27	21.0, 15–26	0.0, -3-9	0.752	0.072
FAB (/18)	13.0, 9–15	15.0, 11–16	2.0, -1-4	0.006	11.0, 5–16	10.0, 4–14	-1.0, -2-2	0.279	0.007
RAVLT									
Immediate recall (/75)	25.0, 14–41	28.0, 14–43	3.0, -6-8	0.035	18.0, 6–24	17.0, 5–22	-1.0, -4-2	0.288	0.011
Delayed recall (/15)	2.0, 0–9	5.0, 0–10	2.0, -1-4	0.008	0.0, 0–3	0.0, 0–4	0.0, -1-2	0.414	0.122
Recognition (/30)	27.0, 20–30	28.0, 22–30	1.0, -2-4	0.026	21.0, 14–25	18.0, 14–27	-1.0, -4-4	0.527	0.037
TUG									
Completion time (seconds)	12.4, 7.2–53.7	10.1, 7.0-20.4	-1.6, -33.5-1.4	0.011	16.9, 8.4–50.2	12.3, 9.0-22.4	-1.7, -27.8-2.6	0.128	0.906
Number of steps	22.0, 14–81	19.0, 14–40	-3.0, -49.0-0	0.005	21.0, 16–62	19.0, 17–30	-32.0-2	0.102	0.968

Data are presented as medians and ranges. Wilcoxon signed-rank tests and Mann-Whitney U tests were used to evaluate the changes in the clinical measures after shunt placement between the preserved and reduced DMN connectivity groups and within each patient group, respectively

*DMN* default mode network, *FAB* frontal assessment battery, *iNPH* idiopathic normal pressure hydrocephalus, *iNPHGS* idiopathic normal pressure hydrocephalus grading scale, *MMSE* Mini-Mental State Examination, *Post-op* post-operative, *RAVLT* Rey auditory verbal learning test, *TUG* Timed Up and Go test

### Neuropsychological tests for predicting relative DMN connectivity

The ROC curves of the iNPHGS cognition subscores and the RAVLT immediate recall, delayed recall and recognition scores in differentiating the preserved DMN connectivity group from the reduced DMN connectivity group were drawn. The areas under the ROC curves were as follows: iNPHGS cognition subscore, 0.780; RAVLT immediate recall score, 0.857; RAVLT delayed recall score, 0.775; and RAVLT recognition score, 0.890. The best predictor for distinguishing the preserved and reduced DMN connectivity groups was the RAVLT recognition score. The ROC curve of the RAVLT recognition score yielded an optimal cut-off value of 23.5, where the sensitivity was 84.6 % and the specificity was 85.7 %.

## Discussion

In the present study, we investigated DMN connectivity and brain white matter involvement in patients with iNPH. In addition, we examined the differences in clinical characteristics between the patients who showed relatively preserved DMN connectivity and those who showed reduced DMN connectivity relative to the severity of brain white matter disruption. The two major findings of the study were as follows: (1) patients with reduced DMN connectivity relative to the severity of brain white matter disruption exhibited more severe memory impairments at baseline and poorer improvements in executive and memory functions after shunt placement than those with relatively preserved DMN connectivity; and (2) the RAVLT recognition score yielded the highest sensitivity and specificity in differentiating patients with relatively preserved DMN connectivity from those with relatively reduced DMN connectivity.

### DMN connectivity in patients with iNPH

In our study, DMN connectivity in patients with iNPH was lower than that in HCs. This finding is consistent with the results of Khoo et al.’s study [27]. However, DMN connectivity was positively correlated with FAB and RAVLT immediate recall scores and was negatively correlated with iNPHGS cognitive subscores in our study, which indicated that preserved DMN connectivity was linked to better cognitive performance. Surprisingly, these trends are in contrast to the results of Khoo et al.’s study [27]. Khoo et al. postulated that DMN connectivity might decrease to compensate for impaired cognition, attention, gait, and continence in patients in the mild iNPH stage because the DMN has been considered a “task-negative” network [24]. They also speculated that as patient conditions deteriorate in the severe iNPH stage, there is a breakdown of the compensatory decrease in DMN connectivity that might result in an increase in DMN connectivity relative to that in the mild iNPH stage. The differences between these two studies were that the patients in our study were older and exhibited slightly milder cognitive impairments than those in Khoo et al.’s study. One possible explanation for the discrepancy in findings between studies is that compensation due to a decrease in DMN connectivity may not occur in very mild iNPH stages. However, the distributions of cognitive scores in our patients almost overlapped with those in Khoo et al.’s study. Moreover, there has been no report that global DMN connectivity increased with age in elderly individuals [47, 49]. Another possible reason is the poor test-retest reproducibility for measuring DMN connectivity in patients with iNPH. The group-ICA approach for identifying DMN connectivity has been reported to be reliable and valid [50, 51]. However, a recent study indicated that the test-retest variability in resting-state networks increases with age and cognitive decline, and motion during MRI scanning is a confounding factor for increased variability [52]. Thus, cognitive impairment, age, and/or motion during MRI scanning in patients with iNPH might cause poor test-retest reproducibility. Future large-scale cohort studies are needed to elucidate the causes of the discrepancy in the results.

### iNPH patients with relatively reduced DMN connectivity

The iNPHGS cognitive subscores and the RAVLT immediate recall, delayed recall and recognition scores were significantly worse in the reduced DMN connectivity group than in the preserved DMN connectivity group at baseline. However, there was no significant difference in the FAB scores between the two groups. These findings revealed the presence of severe memory disturbances in the reduced DMN connectivity group and were consistent with our hypothesis. The proportion of iNPH patients with reduced DMN connectivity relative to the severity of brain white matter was 35 % in the present study. Several previous studies involving cortical biopsy for patients with iNPH reported that AD pathology was present in 25-67.6 % of patients with iNPH [11, 53, 54]. Although a small number of patients with iNPH were included in our study, the proportion of iNPH patients with relatively reduced DMN connectivity overlapped with the proportion of patients presenting with AD pathology. In addition, DMN connectivity was not associated with whole-brain white matter integrity in the patients with iNPH. This finding indicated that the reduced DMN connectivity in patients with iNPH was not mainly attributable to white matter involvement. Thus, the reduced DMN connectivity relative to brain white matter involvement in patients with iNPH seems to be associated with the presence of AD pathology even though we did not evaluate CSF biomarkers for AD or perform amyloid imaging. Moreover, the reduced DMN connectivity group showed poorer improvements in the RAVLT immediate recall and recognition scores and FAB scores after shunt placement than the preserved DMN connectivity group. Hamilton et al. reported that patients with iNPH with moderate-to-severe tau and Aβ pathology showed diminished postoperative cognitive and motor improvements relative to patients lacking AD pathology [11]. The poor cognitive outcomes after shunt placement among the patients with relatively reduced DMN connectivity in our study was also indicative of an association between relatively reduced DMN connectivity and the presence of AD pathology.

In contrast, the iNPHGS gait subscores were significantly improved after shunt placement in the reduced DMN connectivity group. This finding indicated that shunt placement was effective for improving gait disturbance not only for patients with relatively preserved DMN connectivity but also for patients with relatively reduced DMN connectivity. Although previous studies have demonstrated that gait disturbance in patients with AD is associated with cognitive dysfunction, such as deficits in attention and executive dysfunction, our findings suggested that the reduced DMN connectivity was not apparently related to the severity of the gait disturbance because there was no significant difference in gait function between the preserved and reduced DMN groups at baseline [55]. Therefore, it is thought that gait disturbance in patients with iNPH is mainly caused by its corresponding pathological mechanism.

### Neuropsychological tests for predicting relative DMN connectivity

Among the clinical measures we examined, the RAVLT recognition score was the best measure for differentiating iNPH patients with relatively preserved DMN connectivity from those with reduced DMN connectivity relative to the severity of brain white matter disruption. Although RAVLT recognition scores were not significantly correlated with DMN connectivity among patients with iNPH, the difference in RAVLT recognition scores between the iNPH patients with relatively preserved DMN connectivity and those with relatively reduced DMN connectivity was the most statistically significant among the clinical measures we examined. The results of previous studies on memory function in patients with iNPH are inconsistent. Ogino et al. reported that memory and orientation in patients with iNPH were more preserved than in those with AD, while Saito et al. demonstrated that both recognition and recall memory were impaired in patients with iNPH and in patients with AD [37, 56]. Based on Hamilton et al.’s and Kazui et al.’s studies, it may be that these conflicting results reflect the presence of AD pathology [11, 12]. Unfortunately, because Ogino et al.’s and Saito et al.’s studies did not investigate shunt responsiveness in patients or memory function in shunt non-responsive patients, these studies could not clarify whether the memory deficits at baseline were related to poor cognitive outcomes. However, several previous studies with patients with iNPH reported that patients with memory deficits at baseline were less likely to show cognitive improvement and that memory deficits at baseline were the most significant predictor of dementia after shunt placement [57, 58]. Thomas et al. also reported that poor verbal immediate memory was strongly associated with poor cognitive outcomes after shunt placement. Although the RAVLT immediate recall score in the current study was the second-best predictor of relative DMN connectivity in patients with iNPH, the area under the ROC curve of the score was almost equal to that of the RAVLT recognition score. Memory recall is affected by attention, which is one of the disrupted cognitive characteristics in patients with iNPH [37, 38]. In particular, attention is thought to affect the success of encoding during the learning phase [59]. Therefore, the combined deficits of memory and attention due to the presence of AD pathology in patients with iNPH might make learning difficult, leading to poor performance in the RAVLT immediate recall test.

### Limitations

Our study has several limitations. First, we did not evaluate CSF biomarkers for AD or perform amyloid imaging because this study was a single hospital-based study. Therefore, we could not directly prove that the reduced DMN connectivity relative to the severity of brain white matter disruption was derived from the presence of AD pathology in iNPH patients. Second, we enrolled a small number of patients with iNPH and HC controls in the present study. Therefore, it may not be evident that the relatively reduced DMN connectivity in patients with iNPH was associated with poor cognitive outcomes after shunt placement. Future larger-scale studies are needed to confirm our findings. Third, the mean age of the HCs was significantly lower than that of the iNPH patients. Previous studies have suggested that DMN connectivity tends to decline with ageing [49]. Therefore, we might have overestimated the decrease in DMN connectivity and cognitive impairments in the patients with iNPH.

## Conclusions

Our study demonstrated that for iNPH patients, reduced DMN connectivity relative to the severity of brain white matter disruption is associated with severe memory deficits at baseline and poorer cognitive outcomes after shunt placement with respect to HCs. Thus, the combined indexes of DMN connectivity and brain white matter integrity may predict poor cognitive outcomes after shunt placement in patients with iNPH. However, future larger-scale studies are needed to confirm these findings. In the future, we will try to elucidate whether the relatively reduced DMN connectivity is related to the presence of AD pathology.

## Supplementary Information

**Additional file 1: Supplementary Table 1.** Demographic and clinical characteristics of each participant.

## Data Availability

The magnetic resonance imaging datasets used and/or analysed during the current study cannot be shared publicly because the data contain personally identifiable information. Public disclosure of the data is restricted by the ethics committees of South Miyagi Medical Center. The datasets are available from the corresponding author upon reasonable request. All other relevant data are within the manuscript and its Additional Material file.

## Abbreviations

AD: Alzheimer’s disease
CSF: Cerebrospinal fluid
DESH: Disproportionately enlarged subarachnoid space hydrocephalus
DMN: Default mode network
DMN/FA: Default mode network connectivity/mean fractional anisotropy value within the brain white matter region of interest
DTI: Diffusion tensor imaging
FA: Fractional anisotropy
FAB: Frontal assessment battery
FOV: Field of view
HCs: Healthy controls
ICA: Independent component analysis
iNPH: Idiopathic normal pressure hydrocephalus
iNPHGS: Idiopathic normal pressure hydrocephalus grading scale
MD: Mean diffusivity
MELODIC: Multivariate Exploratory Linear Optimized Decomposition into Independent Components
MMSE: Mini-Mental State Examination
MRI: Magnetic resonance imaging
RAVLT: Rey auditory verbal learning test
ROC: Receiver operating characteristic
ROI: Region of interest
ROI FA: Mean FA value within the whole-brain white matter region of interest
3D-SPGR: Three-dimensional spoiled gradient echo
TBSS: Tract-based spatial statistics
TE: Echo time
TR: Repetition time
TUG: Timed Up and Go test
VP: Ventriculoperitoneal
