# Supplementary material for: Reduced default mode network connectivity relative to white matter integrity is associated with poor cognitive outcomes in patients with idiopathic normal pressure hydrocephalus
Source: BMC Neurol. 2021 Sep 13;21:353. doi: 10.1186/s12883-021-02389-0 (PMC8436532; doi:10.1186/s12883-021-02389-0)
Supplement: Supplementary file 1 — Additional file 1: Supplementary Table 1. Demographic and clinical characteristics of each participant. [file 12883_2021_2389_MOESM1_ESM.pdf]

Supplementary Table 1. Demographic and clinical characteristics of each participant.

A. Baseline

Registration number	Age	Sex	Disease duration (year)	Education attainment (year)	Shunt responsiveness	iNPHGS Gait (0–4)	iNPHGS Cognition (0–4)	iNPHGS Urination (0–4)	iNPHGS Total (0–12)	MMSE (/30)	FAB (/18)
iNPH01	73	M	1.08	12	SR	2	2	2	6	25	15
iNPH02	82	M	4.00	14	SR	2	2	2	6	27	13
iNPH03	85	M	2.08	6	SNR	2	3	2	7	11	8
iNPH04	77	F	3.92	14	SR	1	2	1	4	25	15
iNPH05	89	M	2.67	8	SR	3	2	1	6	23	15
iNPH06	79	M	0.42	9	SNR	0	2	0	2	23	16
iNPH07	65	M	2.25	12	SNR	2	2	1	5	27	13
iNPH08	79	F	2.67	12	SR	1	2	3	6	27	14
iNPH09	84	F	0.08	8	SNR	2	3	2	8	15	5
iNPH10	82	M	5.08	9	SNR	2	3	3	8	23	8
iNPH11	80	F	1.75	9	SR	3	2	0	5	23	11
iNPH12	80	M	4.58	9	SR	1	2	1	4	24	13
iNPH13	81	F	5.58	9	SR	2	1	1	4	28	14
iNPH14	77	F	0.17	9	SR	2	2	1	5	29	11
iNPH15	84	M	5.08	9	SR	2	2	1	5	25	12
iNPH16	86	M	0.58	8	SR	3	2	3	8	13	11
iNPH17	77	F	2.17	9	SR	1	2	2	5	21	10
iNPH18	86	M	0.58	16	SR	3	3	2	8	20	9
iNPH19	72	M	1.17	18	SNR	1	1	0	2	28	13
iNPH20	69	F	2.25	12	SR	2	3	3	8	27	11
HC01	71	F		12						26	14
HC02	71	M		12						25	14
HC03	79	F		12						24	17
HC04	63	F		12						29	18
HC05	68	F		12						30	17
HC06	72	F		9						29	15
HC07	63	M		12						30	17
HC08	62	F		12						30	16
HC09	68	M		12						29	16
HC10	85	M		16						29	18

Registration number	RAVLT immediate recall (/75)	RAVLT delayed recall (/15)	RAVLT recogntion (/30)	TUG completion time (seconds)	TUG number of steps	DMN connectivity	ROI FA	DMN/FA ratio
iNPH01	21	3	23	18.3	38	2.6381	0.4876	5.4104
iNPH02	27	2	29	15.9	20	2.9987	0.4538	6.6080
iNPH03	8	0	21	9.7	17	1.8870	0.4642	4.0651
iNPH04	41	9	29	7.2	14	3.3089	0.4715	7.0178
iNPH05	25	2	24	24.2	30	3.1664	0.4635	6.8315
iNPH06	19	0	16	8.4	16	2.4574	0.4804	5.1153
iNPH07	25	2	26	11.9	24	2.5122	0.4287	5.8600
iNPH08	19	1	28	12.4	22	3.0598	0.4930	6.2065
iNPH09	12	0	20	21.6	32	2.0212	0.4357	4.6390
iNPH10	6	0	14	16.9	20	2.0742	0.4749	4.3677
iNPH11	18	2	22	50.2	62	2.2966	0.4794	4.7906
iNPH12	20	1	21	8.1	15	3.5704	0.4790	7.4539
iNPH13	39	4	30	11.7	23	2.8556	0.4371	6.5331
iNPH14	30	4	27	12.4	21	2.9441	0.4486	6.5629
iNPH15	26	5	27	12.8	26	2.8174	0.4428	6.3627
iNPH16	14	1	20	28.9	65	2.8386	0.4254	6.6728
iNPH17	24	3	28	7.6	15	2.9782	0.4428	6.7258
iNPH18	16	0	25	53.7	81	2.7688	0.4328	6.3974
iNPH19	30	4	25	8.2	15	3.1607	0.4624	6.8354
iNPH20	24	2	25	11.8	21	2.6654	0.4670	5.7075
HC01	39	9	29			5.7058	0.4914	11.6113
HC02	28	7	27			2.5753	0.4961	5.1911
HC03	32	8	27			1.9579	0.4715	4.1525
HC04	49	13	27			5.2062	0.4871	10.6882
HC05	37	8	27			3.2782	0.4810	6.8154
HC06	42	9	27			3.3361	0.4650	7.1744
HC07	35	8	29			4.4697	0.4797	9.3177
HC08	54	11	30			4.3393	0.4717	9.1993
HC09	47	9	28			3.1222	0.4698	6.6458
HC10	30	6	28			4.5759	0.5002	9.1481

B. 6 months after shunt placement

Registration number	iNPHGS Gait (0–4)	iNPHGS Cognition (0–4)	iNPHGS Urination (0–4)	iNPHGS Total (0–12)	MMSE (/30)	FAB (/18)	RAVLT immediate recall (/75)	RAVLT delayed recall (/15)	RAVLT recognition (/30)	TUG completion time (seconds)	TUG number of steps
iNPH01	1	2	1	4	26	14	22	4	27	11.4	21
iNPH02	2	1	1	4	28	15	31	6	28	17.3	20
iNPH03	1	3	3	7	20	7	8	0	18	12.3	19
iNPH04	1	1	1	3	30	16	43	10	30	7.0	14
iNPH05	2	2	1	5	25	15	28	4	26	17.0	24
iNPH06	0	2	0	2	21	14	17	0	15	9.0	17
iNPH07	2	2	1	5	28	13	26	2	27	12.2	22
iNPH08	1	2	2	5	28	13	17	2	26	9.6	16
iNPH09	2	3	2	8	16	7	12	0	20	20.8	30
iNPH10	2	3	3	8	21	8	5	0	14	14.1	18
iNPH11	2	2	0	4	24	13	20	4	24	22.4	30
iNPH12	1	2	0	3	28	15	14	0	22	8.0	15
iNPH13	1	1	1	3	28	15	42	6	30	10.1	19
iNPH14	1	2	0	3	29	15	34	6	29	10.1	18
iNPH15	1	2	1	4	27	14	28	5	29	10.6	22
iNPH16	2	2	2	6	18	12	16	3	24	20.4	40
iNPH17	1	2	1	4	23	12	27	5	29	7.0	14
iNPH18	2	2	2	6	24	11	20	0	27	20.2	32
iNPH19	0	1	0	2	29	15	38	6	28	7.0	14
iNPH20	1	2	3	6	27	14	28	3	27	10.1	19

DMN, default mode network; DMN/FA, default mode network connectivity/mean fractional anisotropy value within the brain white matter region of interest; FA, fractional anisotropy; FAB, Frontal Assessment Battery; HC, healthy control; iNPH, idiopathic normal pressure hydrocephalus; iNPHGS, idiopathic normal pressure hydrocephalus grading scale; MMSE, Mini-Mental State Examination; RAVLT, Rey Auditory Verbal Learning Test; ROI FA, mean FA value within the whole-brain white matter region of interest; SNR, shunt non-responder; SR, shunt reponder; TUG, Timed Up and Go test.
